# “Goals in Focus”—a targeted CBT approach for motivational negative symptoms of psychosis: study protocol for a randomized-controlled feasibility trial

**DOI:** 10.1186/s40814-023-01284-4

**Published:** 2023-05-02

**Authors:** Alisa L. A. Schormann, Matthias Pillny, Katharina Haß, Tania M. Lincoln

**Affiliations:** Clinical Psychology and Psychotherapy, Institute of Psychology, Faculty of Psychology and Human Movement, Universität Hamburg, Von-Melle-Park 5, 20146 Hamburg, Germany

**Keywords:** Cognitive behavioral therapy, Goal-focused, Amotivation, Motivational negative symptoms, Experiential negative symptoms, Anhedonia, Trial protocol, Feasibility, Acceptability

## Abstract

**Background:**

The reduction of goal-directed behavior is the main characteristic in motivational negative symptoms of psychosis as it accounts for the long-term decline in psychological well-being and psychosocial functioning. However, the available treatment options are largely unspecific and show only small effects on motivational negative symptoms. Interventions that directly target the relevant psychological mechanisms are likely to be more effective. For “Goals in Focus”, we translated findings from basic clinical research on mechanisms underlying motivational negative symptoms into a tailored and comprehensive novel psychological outpatient treatment program. With this study, we will test the feasibility of the therapy manual and the trial procedures. We also aim to examine first estimates of the effect size that can be expected from “Goals in Focus” to inform the sample size calculation of a subsequent fully powered trial.

**Methods:**

Thirty participants diagnosed with a schizophrenia spectrum disorder and at least moderate motivational negative symptoms will be randomly assigned to either 24 sessions of “Goals in Focus” over the course of 6 months (*n* = 15) or to a 6-month wait-list control group (*n* = 15). Single-blind assessments will be conducted at baseline (*t*_0_) and 6 months after baseline completion (*t*_1_). Feasibility outcomes include patient recruitment, retention, and attendance rates. Acceptability will be rated by trial therapists and by participants at end of treatment. Primary outcome for effect size estimation is the motivational negative symptom subscale sum score of the Brief Negative Symptom Scale at *t*_1_ corrected for baseline values. Secondary outcomes include psychosocial functioning, psychological well-being, depressive symptoms, expressive negative symptoms, negative symptom factor scores, and goal pursuit in everyday life.

**Discussion:**

The feasibility and acceptability data will be used to improve trial procedures and the “Goals in Focus” intervention where necessary. The treatment effect on the primary outcome will provide the basis for the sample size calculation for a fully powered RCT.

**Trial registration:**

1) ClinicalTrials.gov, NCT05252039. Registered on 23 February 2022.

2) Deutsches Register Klinischer Studien, DRKS00018083. Registered on 28 August 2019.

**Supplementary Information:**

The online version contains supplementary material available at 10.1186/s40814-023-01284-4.

## Background

Apathy, anhedonia, and social withdrawal constitute the motivational factor of negative symptoms [1, 2] and are evident in approximately 60% of people with psychotic disorders [3]. Compared to positive symptoms and the expressivity factor of negative symptoms (i.e., alogia and blunted affect), motivational negative symptoms account for the reduced levels of long-term functioning [4, 5] and the low quality of life [6, 7]. Consequently, both clinicians [8] and patients [9] consider motivational negative symptoms an important treatment target.

Several meta-analyses found that psychological approaches, such as cognitive behavior therapy (CBT), social skills training, and cognitive remediation, can alleviate motivational negative symptoms, however, with only small to moderate effect sizes [10-14]. Reasons for these rather unsatisfying effect sizes could be that the available interventions were either not derived from empirical knowledge about negative symptoms and that each intervention targets only single factors, e.g., social skills [15], beliefs [16], or goal pursuit [17, 18]. Also, emerging qualitative research has emphasized the importance of user involvement in the development of interventions for psychosis [19, 20]. Meanwhile, empirical research on psychological mechanisms of motivational negative symptoms has gained momentum. This has considerably refined and extended our understanding of the factors that are likely to be relevant to the formation and maintenance of negative symptoms. This research now provides a comprehensive set of factors that can be, and in our view *should* be, addressed by psychological interventions. As we will outline in the following, these include goal setting, altered reward processing, anticipatory anhedonia, demotivating beliefs as well as reduced social and problem-solving skills (for an overview see [21]).

Goal setting. Even though people with negative symptoms set personal life goals [22], they report difficulties initiating and maintaining behavior towards goal realization [23]. More fine-grained analyses of short-term goals in a subclinical sample in daily life indicate an association between negative symptoms and setting too many avoidance goals [24]. This is likely to explain the reduced approach-oriented [25] and increased avoidance-oriented behavior [26]. In addition to these internal challenges, several external challenges, such as social deprivation [27] and stigmatization [28, 29], may render it more difficult to set approach-focused goals in this population [30]. Thus, effective psychosocial interventions aimed at optimizing goal pursuit ideally need to address both internal and external challenges. This could be done by encouraging the setting of personally meaningful, specific, measurable, attractive, realistic, and time-bound approach goals that are also suited to optimize the individuals’ external social situation.

Altered reward processing. People with motivational negative symptoms have also been found to display problems in reinforcement learning [26, 31, 32], prediction of reward cues, generating, updating, and maintaining value representations [33-36], exploratory behaviors with uncertain reward-outcomes [37], and unfavorable trade-offs in effort-value computations [5, 33, 34, 38]. Intervention targets that can be derived from this research on reward processing include training the mental representation of a reward and developing an accurate estimation of the effort necessary to achieve a goal.

Anticipatory anhedonia. Recent reviews point towards a reduced ability to anticipate pleasure for future events in people with negative symptoms [39, 40], despite intact ability to experience in-the-moment pleasure [41]. This problem in anticipatory pleasure has been found to mediate the translation of goal intentions into goal-directed behavior [42]. Research from basic neuroscience has found that the anticipation of positive future events draws strongly on the ability to recall pleasant episodic memories [43]. However, this ability has also been found to be reduced in people with negative symptoms [44, 45]. Taken together, this research indicates that improving anticipatory anhedonia could be a key to improve motivation. This could be achieved by supporting the recall of episodic memories about similar past pleasurable experiences [46] and by building on the intact ability to experience consummatory pleasure [47, 48].

Demotivating beliefs. Demotivating beliefs about self (self-defeating beliefs), others (social indifference beliefs), and the future (low expectancy of pleasure) (see [49-52]) have been found to account for one third of variance in amotivation [44, 52] and to impede the willingness to exert effort [53]. Particular attention has been given to a specific aspect of self-defeating beliefs, namely, defeatist performance beliefs (e.g., “If you cannot do something well, there is little point in doing it at all.”) that are associated with a reduced level of functioning [54]. It follows that identifying, challenging, and gradually modifying these specific beliefs could be another promising treatment approach.

Reduced interpersonal and problem-solving skills. Impaired social skills and social cognition have long been associated with motivational negative symptoms [55, 56]. Targeting social skills has been shown to improve negative symptoms [57-59]. Similarly, difficulties in the ability to solve problems in an effective and timely manner have been repeatedly found to be associated with psychosis in general [60] and negative symptoms specifically [61]. Training of problem-solving skills has also become a well-established approach in the treatment of psychosis [62, 63]. Accordingly, effective interventions for people with negative symptoms would be advised to include a focus on problem-solving skills and to encourage the translation of these skills into daily life.

We argue that each of these factors contributes to the observed difficulties of patients with negative symptoms in pursuing personally meaningful goals. It follows that interventions that specifically target these factors are likely to be more successful in reducing motivational negative symptoms than less specific interventions, such as behavioral activation and cognitive therapy. Given that each of these factors is associated with negative symptoms but none of them can fully account for negative symptoms, we also expect approaches addressing several of these factors to be superior to interventions that focus on a single factor. This expectation is corroborated by the fact that approaches that have targeted several of these factors, e.g., by combining cognitive interventions with social skills training [64] or with interventions targeting anhedonia [65-67], tend to produce more promising effects. In addition, given that the factors driving amotivation might vary between patients, we argue that intervention approaches will more likely be effective if their focus can be flexibly adjusted to the individual needs of a given patient [21]. However, to our knowledge, there is no approach that addresses the full range of relevant mechanisms in a customizable manner.

On that account, we developed a novel comprehensive, individualized cognitive behavioral outpatient intervention (“Goals in Focus”) that supports patients in specifying attainable personal goals and overcoming obstacles in goal pursuit. The intervention aims at supporting patients to realize personally meaningful goals by addressing reward processing, anticipatory pleasure, demotivating beliefs, and social and problem-solving skills.

With this feasibility trial, we aim to test the feasibility (i.e., referrals, eligibility rate, number of consenting participants, retention rate, attendance rate, dose of intervention, data attrition, and treatment adherence) and acceptability (i.e., drop-out rate, number of adverse events, participants’ and therapists’ satisfaction) of the therapy manual and trial procedures as well as to obtain first estimates of the effect size that can be expected from “Goals in Focus” (i.e., extent of change in motivational negative symptoms as primary outcome) to inform the sample size calculation for a fully powered clinical trial.

## Methods

This trial protocol was developed in accordance with the Consolidated Standards of Reporting Trials (CONSORT) and the Standard Protocol Items: Recommendations for Interventional Trials (SPIRIT) 2013 Statement [68, 69]. For the SPIRIT checklist, see supplement S1.

### Service user involvement

Two service users and two therapists tested a first version of “Goals in Focus” before this pilot trial and provided feedback on the intervention. The adaptations suggested by the service users were to switch the order of instruments within the assessment phase and to start with the biographical anamnesis, followed by the situational analyses. These suggestions were discussed with the research team and implemented in the current version of “Goals in Focus”. For the current trial, a qualitative user survey will be used at the end of treatment to collect participants’ suggestions for improvement of “Goals in Focus”.

### Study design

**Fig. 1:**
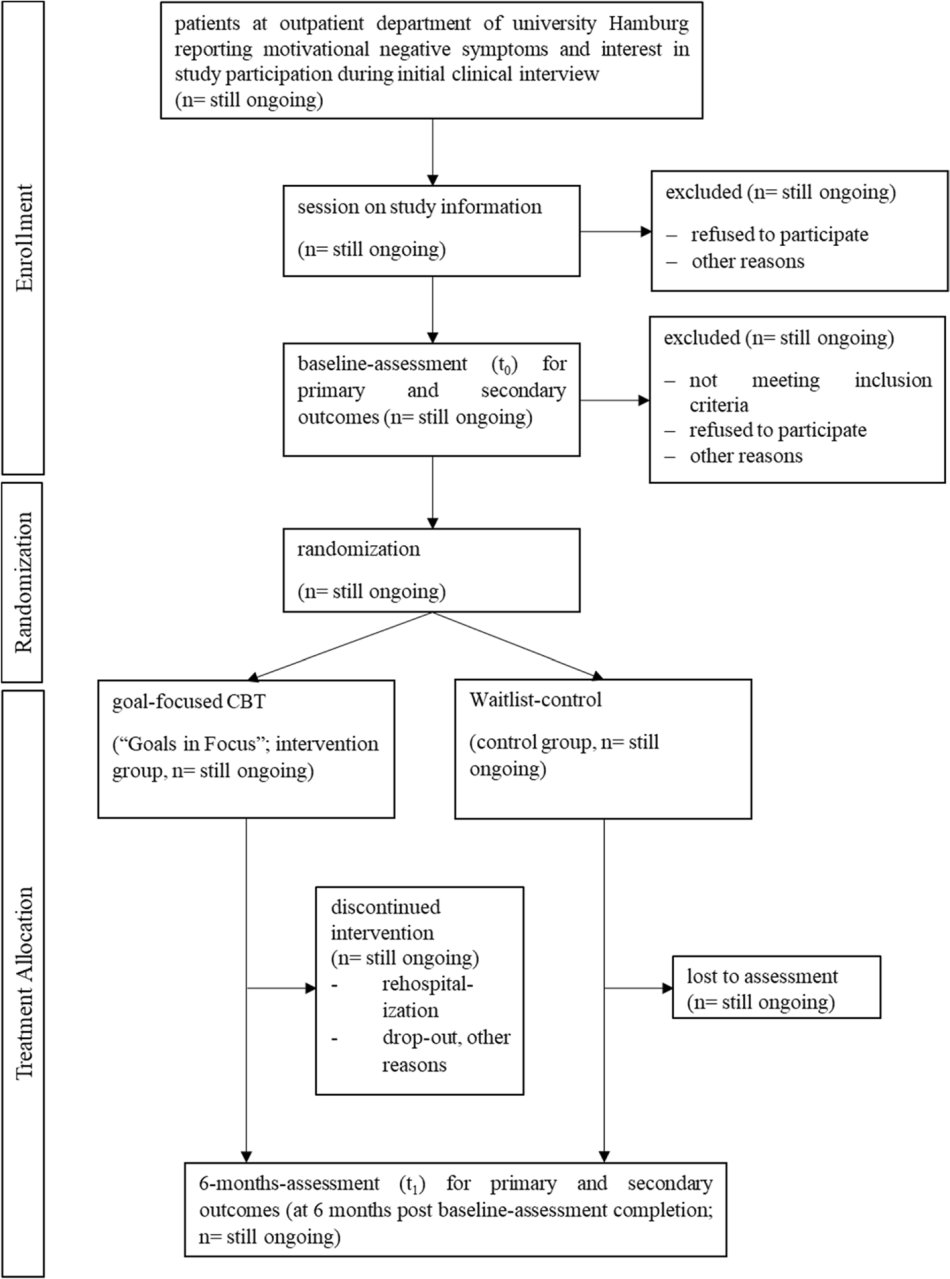
Trial flow diagram

The study is conducted as a single-blind, parallel-group, randomized wait-list controlled feasibility trial (see Fig. 1 for study plan and assessment schedule) implemented at the psychology outpatient clinic at Universität Hamburg. The trial received approval from the ethics committee of the chamber of psychotherapists Hamburg (03/2020-PTK-HH) and has been preregistered at ClinicalTrials.gov (Identifier: NCT05252039) and Deutsches Register Klinischer Studien (Identifier: DRKS00018083). Any modifications to the protocol will be logged using these identifiers and, if required, will be communicated with the participants and secured by amendments to ethical approval. Outcomes will be measured at baseline (*t*_0_) and 6 months after baseline assessment (*t*_1_). Table 1 provides an overview of the trial design and assessment instruments. Recruitment started in 2020 and is planned to terminate in autumn 2023.

**Table 1:**
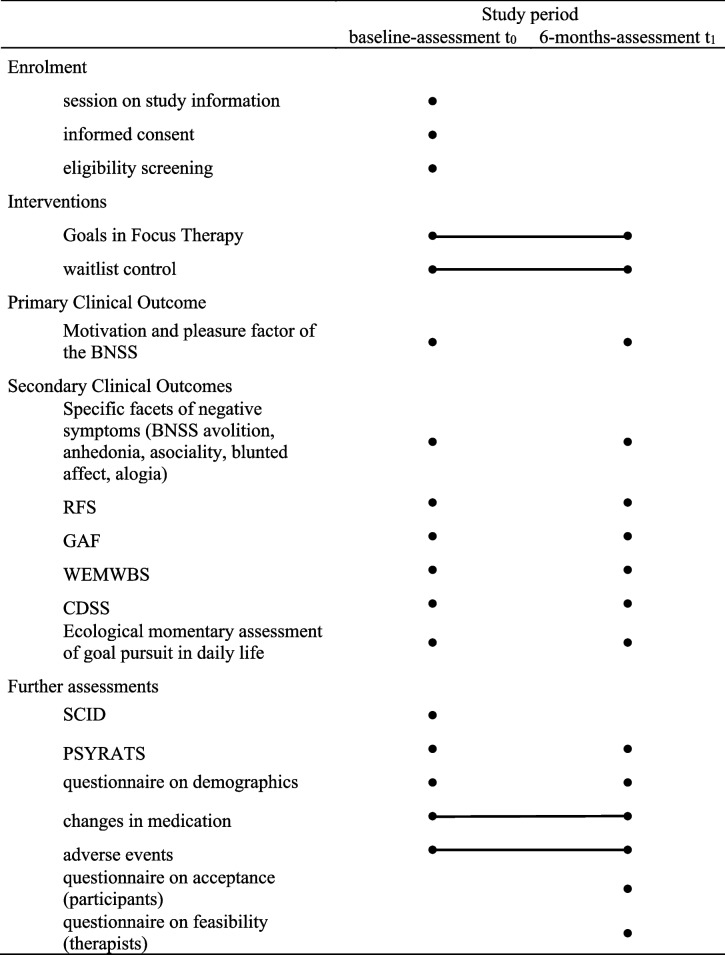
Schedule of enrolment, interventions, and measures at baseline- and 6-months-assessment (SPIRIT guidelines, 2013) [62, 63]

*BNSS* Brief Negative Syndrome Scale, *RFS* Role Functioning Scale, *GAF* Global Assessment of Functioning Scale, *WEMWBS* Warwick–Edinburgh Mental Well-Being Scale, *CDSS* Calgary Depression Rating Scale for Schizophrenia, *SCID* Structured Clinical Interview for DSM-5, *PSYRATS* Psychotic Symptoms Rating Scales

### Participants

#### Eligibility criteria

Eligible participants: (1) meet a diagnosis of a schizophrenia spectrum disorder (confirmed by the Structured Clinical Interview for DSM-5 Clinical Version (SCID-5-CV [70], German version [71]); (2) report at least moderate motivational negative symptoms, i.e., scores ≥ 3 (“moderate”) in two items or ≥ 4 ( “moderately severe”) in one item of the “motivation and pleasure factor” of the Brief Negative Symptom Scale (BNSS) [72, 73]—cut-off criterion was selected based on previous studies with comparable design [18, 74]; (3) be aged between 16 and 85 years; (4) have sufficient skills in German language to participate in psychological therapy (no requirement to speak German fluently); (5) be able to engage in weekly therapy sessions of 50 min; (6) prioritize the reduction of negative symptoms as their current goal for treatment, and (7) be able to give informed consent to participate in the trial. Participants are excluded if they (1) are an immediate and serious risk of harm to themselves or others; (2) have a comorbid diagnosis of alcohol or substance use disorder; (3) report intake of Benzodiazepines for >2 days per week (given that potential side-effects, such as drowsiness and affect motivation), or (4) currently receive any other therapy for motivational negative symptoms.

#### Recruitment, randomization, and blinding

Participants are recruited from outpatient settings in Hamburg (i.e., clinical psychologists and psychiatrists in private practice, social services), psychiatric clinics, and local self-help groups. Participants are either referred to the study by practitioners or via self-referral.

Enrolled and potentially eligible participants are invited for a study information meeting with a trial therapist. The participant will receive a copy of the participant information sheet which provides detailed information about the aims of the study and trial procedures. Participants are given the opportunity to ask questions. Following the information meeting, participants have sufficient time (24 h to 1 week) to consider their participation. After providing informed consent, participants meet with a trial rater who assesses eligibility and completes the baseline assessment with eligible participants. Clinical interviews are conducted by trial raters (psychologists in clinical training with at least master’s degree). All raters have already undergone training in basic therapeutic skills, such as assessment and building rapport. All raters receive a 4-h rater training by the trial manager on all of the observer-rated measures used in this trial. Allocation to either treatment- or wait-list control group is conducted (1:1) using permuted blocks of 4, 6, and 10 to reduce predictability of the sequence. The randomization schedule is generated and preserved by an assistant of the work group who is not otherwise involved in trial procedures. The trial manager is informed about the participant’s group allocation and notifies the participant and the responsible trial therapist.

Blinding of participants is not possible due to the nature of this study. Because trial raters work in the same facilities as trial therapists (i.e., they attend weekly team meetings, meet patients on the hallway, etc.), they also cannot be reliably blinded. Therefore, each assessment is video-recorded and rated by an independent second rater blinded to group allocation and time of assessment. At the beginning of each assessment, participants are reminded to not disclose their group allocation. Video sequences that could reveal the participant’s allocation or point of assessment are deleted before the secondary rating. Secondary raters are asked to record any breaks of blinding, even if the break is equivocal. Putatively unblinded raters are asked to guess the allocation group for the respective participant. Confirmed breaks in blinding of raters follow a standard operating procedure to maintain blind outcome assessments by reallocating “blind” raters for the respective secondary rating, therefore not biasing results.

#### Sample size

An a priori power calculation is not appropriate for this feasibility trial as the study aim is not to test efficacy. Nonetheless, we aim to examine first effect size estimates of “Goals in Focus” on our primary and secondary outcomes. These estimates will inform the sample size calculation for a future fully powered RCT. Based on the recommendations for feasibility studies [75] and on existing feasibility trials in this field [76, 77], the target for this feasibility study was set at *N* = 30 participants (*n* = 15 intervention group; *n* = 15 wait-list control group).

### Interventions

#### Explanation for the choice of comparators

A two-arm, randomized wait-list controlled design was chosen to control for spontaneous remission and to test whether “Goals in Focus” is superior to wait-list control in reducing motivational negative symptoms in patients with psychosis.

#### Wait-list control group

Participants randomized to the “wait-list control group” will be contacted again after 6 months for post-assessment and then offered the “Goals in Focus” intervention. These participants will also be informed that they may use other pharmacological or psychosocial mental health care services but are asked to refrain from psychological interventions that directly target motivational negative symptoms. At the end of the wait-list period, participants in this condition are asked to provide information on any inpatient or outpatient treatment they have received and on major events they consider to have influenced their symptoms during this period.

#### Goals in Focus

**Fig. 2:**
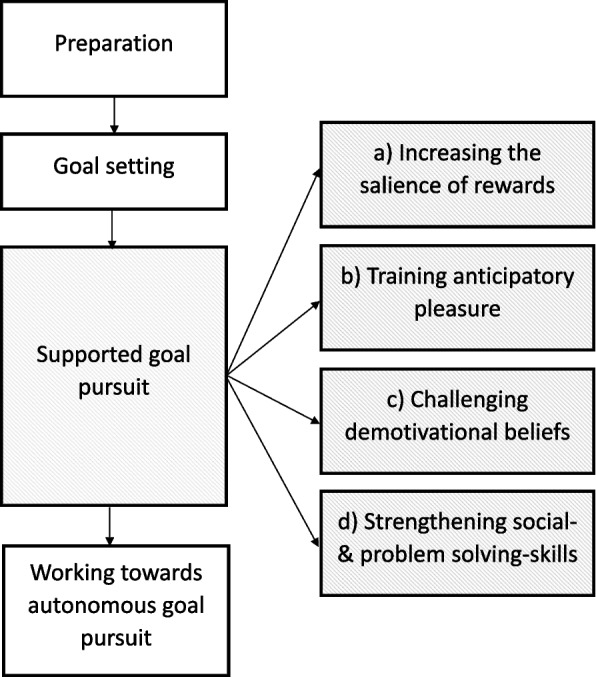
Structure and sequence of “Goals in Focus” intervention

Participants randomized to the “intervention group” will receive up to 24 weekly 50-min sessions of “Goals in Focus” within the following 6 months. The main components of “Goals in Focus” were selected based on basic research [21] and existing therapy approaches on motivational negative symptoms [47, 66, 74, 78]. They were then combined to a customized and readily conveyable manualized approach. “Goals in Focus” follows cognitive–behavioral principles and aims to alleviate motivational negative symptoms by improving goal pursuit. The intervention is delivered in a one-to-one setting by clinical psychologists (master’s degree plus advanced or completed clinical training). All trial therapists receive an additional 2-day training in using the treatment manual, including background information on the rational of “Goals in Focus” and a step-by-step introduction to the individual intervention components. Trial therapists are offered weekly supervision by a senior expert on cognitive behavior therapy for psychosis and are requested to attend at least once per month to increase adherence to the treatment manual. “Goals in Focus” comprises four main phases: (1) preparation, (2) goal setting, (3) supported goal pursuit, and (4) working towards autonomous goal pursuit. An overview on the structure and sequence of “Goals in Focus” intervention is presented in Fig. 2, a table on the domains and intervention elements of “Goals in Focus” is provided in supplement S2.

##### Phase 1, preparation (sessions 1–6)

The aim of phase 1 is to develop an individual case formulation with a focus on the motivational negative symptoms and to collaboratively deduce an individualized treatment plan building on previous experiences of successful and unsuccessful goal pursuit.

##### Phase 2, goal setting (sessions 7–8)

The aim of phase 2 is for participants to set personally valuable goals and to identify first steps towards goal realization. Participants are asked to complete an activity log over the course of 1 week which is used to identify goals that are already being pursued. Next, participants are introduced to different domains of goals (social, recreational, occupational, and health goals) and are asked to select at least one personally meaningful goal related to each of the goal domains. This is complemented by psychoeducation on functional goal setting (e.g., formulating approach goals rather than avoidance goals or cutting long-term goals down into preceding steps of middle- and short term-goals), encouraging patients to adapt these principles to their goals.

##### Phase 3, supported goal pursuit (sessions 9–22)

The goal pursuit phase comprises four consecutive modules of interventions to increase the participants’ goal pursuit. These include (a) increasing the salience of rewards, (b) training anticipatory pleasure, (c) challenging demotivating beliefs, and (d) facilitating social and problem-solving skills. In this phase, the patient is encouraged to set goals that are achievable within 1 week, i.e., until the next session. Aiming to support the pursuit and the achievement of these goals, module a) focuses on reward-processing aiming to improve the representation of the rewards and positive experiences associated with reaching next weeks’ goals. Module b) focuses on improving the anticipation of pleasure with regard to the respective goal by using mental imagery of positive experiences. Module c) focuses on identifying and altering demotivating beliefs that discourage goal pursuit by using cognitive interventions, such as Socratic dialogue or behavioral experiments. Module d) focuses on improving the specific social- and problem-solving skills and aims to build or increase these skills as required for the realization of next week’s goal (e.g., practicing small talk in role play with the therapist in order to accomplish the goal “start a conversation with my neighbor”). Together, participants and trial therapists agree on which of the modules a) to d) will be implemented according to participants’ individual goals and individual problems in goal pursuit.

##### Phase 4, working towards autonomous goal pursuit (sessions 23–24)

The aim of the final phase is to reflect on effective strategies and to prepare for challenges in goal pursuit after therapy termination. Initially set phase 2 goals and the respective goal progress are revised, helpful strategies for goal pursuit are identified and rehearsed, and plans for prospectively pursuing the established or new goals are made.

### Outcomes

#### Feasibility and acceptability measures

To assess feasibility of “Goals in Focus” and trial procedures, the following data is collected: (1) referrals (number of participants referred to the study), (2) eligibility rates (proportion of enrolled participants found eligible), (3) number of participants consenting to study participation and reasons for refusals, (4) retention rate (i.e., number and proportion of participants who attend *t*_1_ assessment and completeness of data at *t*_1_), (5) attendance rates and dose of intervention (i.e., number of sessions delivered within 6 months), (6) data attrition (proportion of outcome data not obtained), and (7) treatment adherence as indicated by the therapists’ self-report in the post-treatment questionnaire.

Acceptability is assessed by (1) drop-out rate (i.e., number of withdrawals), (2) number of adverse events, (3) participants’ satisfaction with treatment and personal outcomes and suggestions for improvement as indicated by each participants’ response to three quantitative and three qualitative questions (see post-intervention questionnaire, supplement S 3; e.g., participants are asked to indicate how helpful they perceived “Goals in Focus” to be for their problems and for approaching their goals and to suggest improvements), and (4) therapists’ satisfaction as measured by the post-intervention questionnaire, therapist version (see supplement S 4).

Progression criteria for a subsequent trial were set according to existing recommendations for feasibility studies [79, 80] and can be found in Table 2.

**Table 2.**
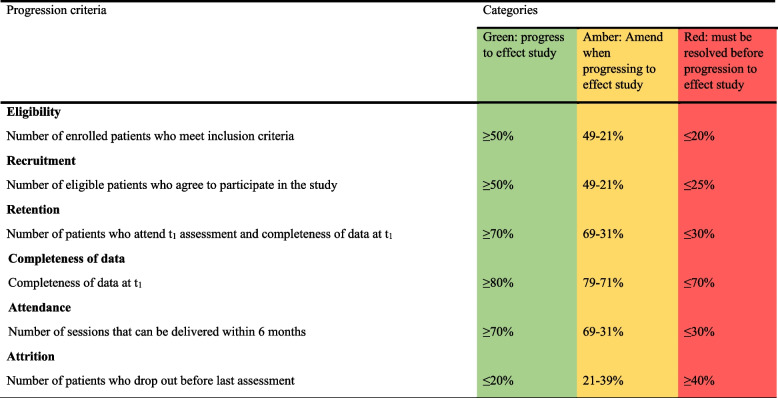
Progression criteria

#### Primary clinical outcome

The primary outcome for effect size estimation is measured with the baseline-adjusted “motivation and pleasure factor” (i.e., sum score of the seven items of this factor) of the Brief Negative Symptoms Scale (BNSS) at *t*_1_ [72, 73]. The BNSS is a 13-item semi-structured interview measuring negative symptoms on two factors, namely “motivation and pleasure” and “reduced expressivity” [81]. The “motivation and pleasure factor” includes seven items that measure the subscales of “anhedonia,” “avolition,” and “asociality” [82]. Symptoms are rated on a 7-point scale ranging from 0 “no impairment” to 6 “severe deficit”.

#### Secondary clinical outcomes

Specific facets of negative symptoms are assessed according to the five-factor model of negative symptoms [83]. This includes the three subscales avolition, asociality, and anhedonia of the BNSS “motivation and pleasure factor” and the two subscales blunted affect and alogia of the BNSS “reduced expressivity factor”.

Psychosocial functioning is assessed using the Role Functioning Scale (RFS) [84] and the Global Assessment of Functioning (GAF) [85]. The RFS is a semi-structured interview and assesses four domains of functioning on the subscales “working productivity,” “independent living,” “immediate social network,” and “extended social network”. Participants’ answers are rated with regard to anchor points, ranging from 1 “low functioning” to 7 “optimal functioning”. For subsequent analyses, a mean score for total functioning will be calculated. The GAF [85] is used as an observer rating of psychosocial functioning. Functioning is rated on a single scale ranging from 1 (lowest) to 100 (highest) based on detailed anchor points.

Subjective well-being is assessed with the Warwick–Edinburgh Mental Well-Being Scale (WEMWBS) [86, 87]. The WEMBS is a self-report questionnaire consisting of 14 statements on well-being during the previous 2 weeks (e.g., “I’ve been feeling good about myself.”). Participants rate these items on a 5-point Likert scale ranging from 1 (“none of the time”) to 5 (“all of the time”).

Symptoms of depression are assessed with the Calgary Depression Rating Scale for Schizophrenia (CDSS) [88, 89]. The CDSS is a 9-item semi-structured interview which has been developed to measure depressive symptoms in patients with schizophrenia. The participant’s answers are rated by the interviewer on a 4-point scale referring to explicit anchor annotations ranging from 0 (“clearly absent”) to 3 (“severe”).

**Fig. 3:**
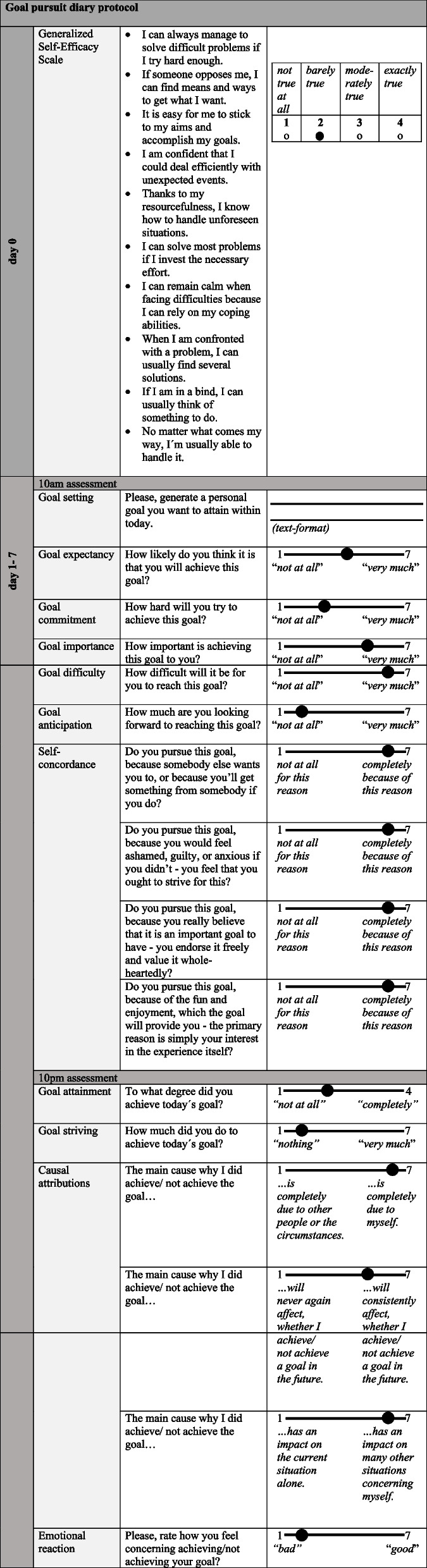
Ecological momentary assessment of goal pursuit in daily life

Goal pursuit in daily life is assessed using ecological momentary assessment (EMA). The EMA protocol can be seen in Fig. 3. Participants will be prompted twice a day (10 a.m. and 10 p.m.) over the course of 1 week via the Movisens XS application (Movisens GmbH) installed on smartphone devices provided by the research team. At the beginning, the participants’ self-efficacy is assessed with the Generalized Self-Efficacy Scale (GSE) [90]. Each of the following “10 a.m. prompts” asks the participants to nominate a goal they would like to achieve during the respective day and to rate characteristics of this goal (e.g., goal commitment, goal importance, goal difficulty, etc.). At the “10 p.m. prompts,” participants are asked to indicate to what extent they achieved the respective goal and to answer questions on their causal attributions and affective experience regarding their goal pursuit.

#### Additional measures

Participants are asked to provide sociodemographic and clinical information (e.g., age, gender, living situation, occupational status, as well as psychiatric history, medication) at baseline. Any changes in medication, occupational status, living situation, or adjunct treatments between *t*_0_ and *t*_1_ are recorded throughout the trial. The severity of positive symptoms is assessed at *t*_0_ and *t*_1_ with the Psychotic Symptom Rating Scales (PSYRATS) [91, 92] to be able to control for a possible confounding influence of reduced positive symptoms on changes in negative symptoms.

### Safety reporting

Any spontaneously reported unfavorable symptom that develops or deteriorates during the study will be classified as an adverse event (AE), whether or not it is considered to be related to the study treatment. AEs comprise exacerbation of pre-existing symptoms, increase in frequency or intensity of a pre-existing episodic event or condition, condition that is detected after trial intervention administration, and continuous persistent illness or a symptom present at baseline that expectedly or unexpectedly worsens following administration of the intervention. Serious adverse events (SAEs) are those considered to be potentially or actually resulting in death, in significant or persistent incapacity/disability, or requiring new or prolonged inpatient hospitalization. The trial team member becoming aware of the AE must record it. The AE is then reviewed by the trial manager and reported to the principal investigator within 72 h to assess causality. In any case of a SAE, the trial manager must be notified within one working day by the member of the trial team becoming aware of the event. The trial manager is required to notify the principal investigator within one working day. The principal investigator is responsible for reporting fatal and life-threatening serious adverse reactions to the competent authorities within 7 days of becoming aware of the event. If necessary due to an adverse event, participants are referred to an inpatient clinic in case of extreme symptom deterioration or substantial risk of harm to self/others by their trial therapist. The trial will be stopped by the principal investigator in case that the rate of AEs attributable to “Goals in Focus” exceeds 30%. Study and treatment participation can be terminated either by the participants themselves at any given time and without justification or by the principal investigator in case of significant harm for the participant. Excluded participants are offered regular treatment at our outpatient clinic. As the trial manager continuously tracks the number of AEs and SAEs throughout the study, no interim safety analysis is planned. The principal investigator and the trial manager have access to the safety data at any given time and will make it available for responsible authorities upon request.

### Data collection, management, confidentiality, and analysis

#### Data collection, management, and confidentiality

The principal investigator has overall responsibility for the trial. The trial manager is responsible for trial procedures, day-to-day data management, and outcome analyses. Only data necessary to conduct the study is obtained. Data is collected by trained trial raters who are supervised by the trial manager. Collected data is handled by the trial manager and kept confidential and stored securely. Physical data (e.g., interview rating sheets) is kept in a locked file cabinet. Digital data (e.g., data obtained via smartphone in ecological momentary assessment) is stored in password-secured and encrypted files which are only accessible by the trial manager. The video recordings of the assessments are stored in encrypted files and will be deleted after completion of data collection. Personal identifiable information will not be released and is stored separately from research data. Research data is pseudonymized. An encrypting list which allows to allocate participant’s names to their trial ID and thus to their data is kept confidential. This list will be destroyed after termination of data collection. Only aggregated and anonymized data will be used for publication. All actions on data management as well as quantitative and qualitative data analyses are carried out by the trial manager and quality checked by the principal investigator. No data monitoring committee is convened for this study due to its short duration and the low risk of harm that can be expected from the intervention.

#### Analysis plan

Feasibility and acceptability will be analyzed by calculating descriptive statistics based on the intention-to-treat (ITT) population. Potential reasons for trial withdrawal will be examined by comparing baseline characteristics (e.g., symptom severity, sociodemographic variables, etc.) between participants who completed the trial and participants who discontinued their participation after randomization.

Clinical outcomes at baseline assessment (*t*_0_) and 6-month assessment (*t*_1_) will be summarized using descriptive statistics (i.e., frequency and percentages on binary and categorical variables; means, standard deviations, or medians, with minimum and maximum values for continuous variables) for each arm of the trial separately. Wait-list control and treatment group characteristics at baseline will be compared using Pearson chi-square tests for the categorical variables and the *t*-test or Mann–Whitney test for the quantitative variables.

Confirmatory analysis will be conducted for primary and secondary outcomes based on the ITT population. An ANCOVA adjusted for baseline values will be calculated with group allocation as between-subject factor and time as within-subject factor. Any variables that show significant group differences at baseline or are found to correlate with the primary outcome (e.g., age, gender, diagnosis, medication doses, duration of illness, symptom severity, etc.) will be entered as covariates. Effect sizes between and within treatment conditions will be assessed by calculating Cohen’s *d*. Missing values will be handled by multiple imputation, if missing completely at random [93]. All analyses will be carried out using validated statistical software.

## Discussion

This trial protocol describes the pilot feasibility trial of the “Goals in Focus” intervention, a new individualized goal-focused form of CBT targeting motivational negative symptoms in people with psychosis. Feasibility and acceptability as well as first estimates of efficacy of “Goals in Focus” will be evaluated and information on possible optimization for a consecutive fully powered RCT will be gathered.

“Goals in Focus” was developed to directly target the relevant motivational negative symptom domains by translating recent empirical research on factors and mechanisms underlying motivational negative symptoms into specified interventions. The comprehensive treatment approach is tested in a randomized controlled trial design which allows to eliminate a possible selection bias and to control for spontaneous remission effects. However, there are some limitations to our study design: this is a pilot trial with the main focus on feasibility and the sample of 30 participants is not sufficient for a reliable efficacy analysis. As likelihood of type 1 error inflation is high, results of significance tests cannot be interpreted, but effect sizes derived from the present study will be used for a priori sample size calculation for a subsequent fully powered RCT. The advantage of a wait-list control condition is that it allows a first estimation of effects and enables wait-list participants to later receive the same intervention. Also, diffusion effects between the conditions (e.g., patients from the wait-list group receiving information about the intervention from other patients, therapists including intervention elements in standard treatment) are unlikely due to the outpatient setting and the fact that treatment as usual is not provided by trial therapists. A disadvantage is that the wait-list comparator may artificially inflate estimates of the intervention effect, due to participants being held in a waiting position and less likely to initiate behavioral change on their own (e.g., as suggested by Cunningham et al. [94]). Additionally, the eligibility criterium of “being able to engage in weekly therapy sessions of 50 min” limits the generalization of effects to those who are not able to attend weekly sessions for the full-time range of 50 min. Other issues that warrant mention relate to the selected outcomes: since “Goals in Focus” aims at improving goal setting and achievement, the EMA of goal pursuit is of particular interest. However, participants’ adherence to an autonomously conducted 1-week survey might be reduced, which is why we did not select it as the primary outcome. Also, a long-term follow-up was not included at this point due to the primary focus being on acceptability, feasibility, and first estimates of efficacy. It also needs to be kept in mind, that the intervention only targets motivational negative symptoms and not expressive negative symptoms, the other relevant component of negative symptoms [72, 95]. However, we expect expressive negative symptoms to be indirectly affected by the intervention as suggested by results reported by Choi et al. [18] in relation to a similar treatment approach. Accordingly, expressive negative symptoms are evaluated as a secondary outcome. Similarly, the intervention does not target contextual factors, such as social deprivation or health-related issues that are likely to exacerbate negative symptoms, but expect the achievement of personal goals due to the intervention to partially improve participants’ challenging contextual factors as well.

If found to be safe, feasible, and acceptable, the “Goals in Focus” intervention will be evaluated in a larger multicenter RCT with pre-post-follow-up design. Thus, this pilot feasibility trial will provide essential information that can be used to optimize the treatment manual, the consecutive design, and the implementation of the fully powered, multi-centered RCT. The further development and evaluation of this intervention can contribute to the much-needed extension of treatment options to support people experiencing motivational negative symptoms in pursuing their goals.

## Trial status

Patient recruitment commenced in 2020 and is scheduled to terminate in 2023 (study protocol—V.1.3, dated November 2021. With protocol submission, 14 participants had been included in the trial. Currently, 22 participants have been included in the trial (*n* = 10 were randomized to “Goals in Focus” and *n* = 12 to the wait-list control group). Dissemination of study results is planned for 2024.

## Dissemination plans

Trial results, including feasibility outcomes, will be disseminated in detail in open-access peer-reviewed scientific publications as well as summarized in consumer-friendly language on the website of Universität Hamburg. Findings will be disseminated to participants and presented at patient events and at local, national, and international conferences.

## Supplementary Information

**Additional file 1: S1.** SPIRIT Checklist. 

**Additional file 2: S2.** Domains and intervention elements of “Goals in Focus”.

**Additional file 3: S3. **“Goals in Focus” t_1_ assessment for participants. 

**Additional file 4: S4. **“Goals in Focus” t_1_ assessment for trial therapists.

## Data Availability

Availability of data not applicable. Material can be obtained upon request from the corresponding author.
