# Supplementary material for: “Goals in Focus”—a targeted CBT approach for motivational negative symptoms of psychosis: study protocol for a randomized-controlled feasibility trial
Source: Pilot Feasibility Stud. 2023 May 2;9:72. doi: 10.1186/s40814-023-01284-4 (PMC10152726; doi:10.1186/s40814-023-01284-4)
Supplement: Supplementary file 2 — Additional file 2: S2. Domains and intervention elements of “Goals in Focus”. [file 40814_2023_1284_MOESM2_ESM.docx]

**S2.**

Domains and intervention elements of “Goals in Focus”

| phases and modules | sessions | key intervention elements |
| --- | --- | --- |
| 1) preparation | 1-6 | biographical anamnesis; situational analyses of patients’ previous experiences of successful and unsuccessful goal pursuit; development of an individual explanatory causal model for motivational negative symptoms; exploration of patients’ level of goal-directed activity in everyday life |
| 2) goal setting | 7-8 | introduction to areas of goal setting (social-, recreational-, occupational-, and health goals); formulation of personally relevant goals; knowledge transfer on how to specify goals; identification of first steps for patients’ goal pursuit |
| 3) goal pursuit | 9-22 | organization of goal pursuit by weekly goal planning; regular reviews and adaptions of initially set goals; keeping track of goal achievement process; identification of obstacles to goal achievement |
| a) increasing the salience of rewards |  | post-processing of goal pursuit (attempts); guiding patients to re-imagine positive emotions (preparation for intervention in anticipatory pleasure); supporting patients to reattribute dysfunctional causal explanations; guiding patients to savor each step on the way to goal achievement by supporting reward planning and encouraging patients to share success and positive emotions with others |
| b) training anticipatory pleasure |  | guiding patients to imagine positive emotions and aspects of planned goal pursuit; implementing the tracking of positive emotions (consummatory pleasure) during goal pursuit |
| c) challenging demotivating beliefs |  | identifying demotivating beliefs; challenging demotivating beliefs; supporting patients to implement new beliefs in sensu and in their everyday life |
| d) strengthening social and problem-solving skills |  | using role play to practice situations relevant to goal pursuit; introducing problem-solving techniques and application to patients’ goal pursuit |
| 4) reflection and preparing for autonomous goal pursuit | 23-24 | reviewing progress/ encouraging patients to reflect on initially set and accomplished goals; discussing patients’ hopes and worries regarding continuation of goal pursuit after therapy; supporting patients in planning for future goal pursuit by drawing on helpful strategies learned during therapy |
