# Supplementary material for: “Goals in Focus”—a targeted CBT approach for motivational negative symptoms of psychosis: study protocol for a randomized-controlled feasibility trial
Source: Pilot Feasibility Stud. 2023 May 2;9:72. doi: 10.1186/s40814-023-01284-4 (PMC10152726; doi:10.1186/s40814-023-01284-4)
Supplement: Supplementary file 3 — Additional file 3: S3. “Goals in Focus” t1 assessment for participants. [file 40814_2023_1284_MOESM3_ESM.docx]

**S3. “Goals in Focus” t_1_ assessment for participants**

We kindly ask you to answer the following questions on the “Goals in Focus” intervention which you completed. Your answers regarding the intervention will contribute to optimization of future treatment options for people with similar difficulties.

| 1. **How helpful do you rate the therapy with regard to your problems?** | | | | |
| --- | --- | --- | --- | --- |
| 🔿  not helpful | 🔿  rather not helpful | 🔿  partly helpful/ partly not helpful | 🔿  rather helpful | 🔿  very helpful |
| 1. **How helpful do you rate the therapy with regard to approach your goals?** | | | | |
| 🔿  not helpful | 🔿  rather not helpful | 🔿  partly helpful/ partly not helpful | 🔿  rather helpful | 🔿  very helpful |
| 1. **How content are you with regard to the therapy you have received?** | | | | |
| 🔿  not content at all | 🔿  rather not content | 🔿  partly content / partly not content | 🔿  rather content | 🔿  very content |
| 1. **What do you consider most helpful about the therapy received?**   ________________________________________________________________________________________________________________________________________________________________________________________________________________________________________________________________________________________________________________________________________________________________________________________________________________________________________________________________________________________________________________________________________________________________________________________________________________________________________________________________ | | | | |
| 1. **What do you consider less helpful about the therapy received?**   ________________________________________________________________________________________________________________________________________________________________________________________________________________________________________________________________________________________________________________________________________________________________________________________________________________________________________________________________________________________________________________________________________________________________________________ | | | | |
| 1. **What do you think needs to be improved about the therapy received?**   ________________________________________________________________________________________________________________________________________________________________________________________________________________________________________________________________________________________________________________________________________________________________________________________________________________________________________________________________________________________________________________________________________________________________________________________________________________________________________________________________ | | | | |
| 1. **What was missing completely in the therapy received?**   ________________________________________________________________________________________________________________________________________________________________________________________________________________________________________________________________________________________________________________________________________________________________________________________________________________________________________________________________________________________________________________________________________________________________________________________________________________________________________________________________ | | | | |
