# Supplementary material for: “Goals in Focus”—a targeted CBT approach for motivational negative symptoms of psychosis: study protocol for a randomized-controlled feasibility trial
Source: Pilot Feasibility Stud. 2023 May 2;9:72. doi: 10.1186/s40814-023-01284-4 (PMC10152726; doi:10.1186/s40814-023-01284-4)
Supplement: Supplementary file 4 — Additional file 4: S4. “Goals in Focus” t1 assessment for trial therapists. [file 40814_2023_1284_MOESM4_ESM.docx]

**S4. “Goals in Focus” t_1_ assessment for trial therapists**

Patient-ID: _______________________

Therapist: _______________________

Date: _______________________

| 1. **How do you rate feasibility of the treatment program?** | | | | | | | | | |
| --- | --- | --- | --- | --- | --- | --- | --- | --- | --- |
| 🔿  very bad | | 🔿  rather bad | | 🔿  partly good/ partly bad | | 🔿  rather bad | | 🔿  very good | |
| 1. **Which interventions or areas of interventions do you consider helpful for your patient?**   ________________________________________________________________________________________________________________________________________________________________________________________________________________________________________________________________________________________________________________________________________________________________________________________________________________________________________________________________________________________________________________________________________________________________________________________________________________________________________________________________ | | | | | | | | | |
| 1. **Which interventions or areas of interventions do you consider not helpful for your patient?**   ________________________________________________________________________________________________________________________________________________________________________________________________________________________________________________________________________________________________________________________________________________________________________________________________________________________________________________________________________________________________________________________________________________________________________________________________________________________________________________________________ | | | | | | | | | |
| 1. **Which feedback on the therapy did you receive from your patient?**   ________________________________________________________________________________________________________________________________________________________________________________________________________________________________________________________________________________________________________________________________________________________________________________________________________________ | | | | | | | | | |
| 1. **Was the treatment discontinued?**   🔿 no  🔿 yes  **🡪 if applicable, what was the reason for discontinuation?**  _____________________________________________________________________________________________________________________________________________________________________________________________________________________________________________________________________________________________________________________________________  **🡪 if applicable, in which session?** ______________________ | | | | | | | | | |
| 1. **Please rate the amount of homework completed by your patient.** | | | | | | | | | |
| 🔿  100%  homework done at all sessions | 🔿  99 - 80 %  homework done at majority of sessions | | 🔿  79 – 60 %  homework done at more than half of the sessions | | 🔿  59 - 40%  homework done at half of the sessions or less | | 🔿  39 – 10%  homework rarely done | | 🔿  9 – 0%  Homework done extremely rarely to never |
| 1. **How close to the treatment manual did you stay?** | | | | | | | | | |
| 🔿  not at all | | 🔿  little | | 🔿  partly | | 🔿  mainly | | 🔿  always | |
| **🡪 if you did not always stick to the treatment manual, what were the reasons for it?**  🔿 insufficient time for sessions` preparation  🔿 not sure on how to implement the interventions  🔿 objectives of patient other than addressed by the manual  🔿 interventions found not fitting, even though patient´s objectives fit the manual focus  🔿 other reasons: _________________________________________________ | | | | | | | | | |
| **🡪 if applicable, what kind of interventions did you use that are not part of the treatment program and why?**  **_____________________________________________________________________________________________________________________________________________________________________________________________________________________________________________________________________________________________________________________________________________________________________________** | | | | | | | | | |
| 1. **Additional comments/ suggestions for improvement**   _______________________________________________________________________________________________________________________________________________________________________________________________________________________________________________________________________________________________________________________________________________________________________________________________________________________________________________________________________________________________________________________________________________________________________________ | | | | | | | | | |
